# Shared decision-making and the lessons learned about decision regret in cancer patients

**DOI:** 10.1007/s00520-021-06725-5

**Published:** 2022-01-15

**Authors:** Mariam Chichua, Eleonora Brivio, Davide Mazzoni, Gabriella Pravettoni

**Affiliations:** 1 Department of Oncology and Hemato-Oncology, University of Milan, Milan, Italy; 2 Applied Research Unit for Cognitive and Psychological Science, IEO, European Institute of Oncology, IRCCS, Milan, Italy

**Keywords:** Shared decision-making, Post-treatment regret, Patient support, Decisional aids

## Abstract

The commentary presents reflections on the literature on post-treatment cancer patient regret. Even though a lot of effort has been made to increase patient satisfaction by engaging them in medical decisions, patient regret remains present in clinical settings. In our commentary, we identify three main aspects of shared decision-making that previously have been shown to predict patient regret. Based on these findings, we provide recommendations for physicians involved in the shared decision-making process. In addition, we make methodological suggestions for future research in the field.

## Introduction

With the increase of cancer treatment options, cancer patients together with their physicians face the challenge to choose among different treatments available to them, considering the risks and benefits of each one. This process is referred to as shared decision-making (SDM) in the literature [1]. Considering the personal values of a patient is central, as it has the potential to increase satisfaction with the treatment and reduce regret [2]. However, despite the efforts to adopt a patient perspective, post-treatment regret within cancer patients remains an issue.

Some background and contextual factors have been previously associated with decisional regret following a cancer treatment like sociodemographic variables, treatment type, impaired physical health, lack of social support, and poor mental health [3]. Moreover, regret has been often considered in negative terms and rarely for its potential implications for the SDM process. Building on the existing literature, in this contribution, we suggest that at least three aspects of the patient-physician decision-making can be conceptually linked to treatment regret: inadequately provided information, the discrepancy between preferred and experienced patient roles, and the physician–patient relationship difficulties.

## Inadequately provided information

The starting point of SDM is informing the patient. Nonetheless, what is the optimal information to be provided? Having enough information about treatments is important to set expectations, but exposure to too much information can be overwhelming [4]. Regret about treatment choice is strongly related to whether patients discussed treatment options with their physicians beforehand and whether they perceived their own decision to be well-informed [5]. Being inadequately informed has been repeatedly reported in retrospect by regretful patients [6, 7]. These findings suggest that healthcare professionals should carefully portray the risks and benefits of the proposed treatment alternatives to form realistic expectations about the post-treatment period. Moreover, to avoid recall bias, it may be more effective to assess information needs and comprehension in the pre-treatment rather than post-treatment phase. Possible assessment tools can be found in Table 1.

**Table 1:** Measurement tools for SDM variables

	Information needs and comprehension	Role preference	Physician–Patient relationship
Measurements	Aid to Capacity Evaluation (ACE) [8] The MacArthur Competence Assessment Tool-Treatment (MacCAT-T) [9]	Control Preference Scale (CPS) [10]	Patient-Doctor Relationship Questionnaire (PDRQ-9) [11]

Aside from the amount of information, the form in which it is presented cannot be overlooked. Medical information, often complex and intimidating, may be challenging to understand for patients. Hence, with an intention to support SDM, decision aids (DAs) are developed to provide patients with explanations about healthcare options. The impact of DAs on decisional regret is still debated; some studies report no effect [12, 13], while others report less patient regret after using DAs [14, 15]. The incongruency in results may reflect the diversity of DAs, which may not have been homogenously designed following quality standards such as the International Patient Decision Aid Standards (IPDAS) when creating DAs [16]. Nonetheless, despite the absence of conclusive findings, some promising results together with the increasing use of DAs in many clinical settings suggest that modifying the form in which information is presented to patients may represent a fruitful strategy to reduce treatment-related regret.

## The discrepancy between the desired patient’s role and the actual one

In SDM discourse, it is believed that an active engagement in the medical pathway decreases decisional conflict and leads to higher patient satisfaction. A large number of studies show that patients actively involved in the decision-making process show significantly lower levels of regret compared to those playing a more passive role[17, 18]; however, there are still others that report exactly the opposite [19, 20].

Perhaps the central point in this debate is to contextualize active and passive roles. While active participation in the decision may give patients the chance to set realistic expectations [18] as well as express their preferences, the health literacy of the patients has to be considered. It has been shown that patients who report too much perceived responsibility have less treatment knowledge and more decision regret [19]. Without adequate clinical recommendations and support, an active role might require that patients undertake greater responsibility than desired, which in turn leads to increased decisional regret, instead of preventing it [20]. Another important contextual factor to consider is culture, and the influence it may have on information disclosure as well as control preference. Results of an international cross-sectional study suggest that education, as well as country of origin, predict decisional control preference [21].

It has been recently suggested [22] that decisional regret is neither associated with the role patients prefer nor with the role they actually adopt. Instead, it is associated with the discrepancy between the two. Involuntary passive role [23], as well as an involuntary active role [20], predicts increased decisional regret. According to this line of thought, an assessment of the patients’ decisional capacity and involvement preferences should be taken into consideration by the clinician, when providing clinical recommendations and support.

## Physician–patient relationship difficulties

Perhaps one of the most “human” aspects of SDM is the quality of the patient-clinician relationship, represented by attitudes and trust they have for one another. Nonetheless, relationship-specific variables are the least investigated ones in the research focusing on decision regret.

Problems with health providers have been listed as one of the common aspects that patients regret in relation to receiving treatment [24]; some of these patients even reported that they would choose another doctor if they could go back in time. Trust in the physician has been recently identified as a strong predictor of decisional regret [25]. Patients who trust their clinicians perceive choosing the treatment as a shared experience and show lower levels of decision regret [26]. Even while experiencing disagreement with the physician, patients report being more confident about the final decision when these disagreements are handled skillfully, with sufficient explanations, availability, and time, provided by an engaged and caring team [26]. Patients’ confidence in oncologists’ consideration of their personal values [27] strongly predicts low levels of decision regret.

Being understood and acknowledged as an individual is important not just for the end result of a medical decision, but as a process within itself.

## Discussion and conclusion

In this contribution, we identified three important aspects of SDM in relation to cancer patient regret. In view of these three aspects, we briefly review the main considerations for healthcare professionals:

1. To facilitate optimal, patient-centered decisions, it is pivotal that patients have an adequate understanding of their treatment options. Providing information about different anti-cancer treatments is recommended, as well as presenting this information in an easy-to-grasp, comprehensive way. The latter may be achieved by communicating the characteristics of each treatment with the help of DAs.
2. Congruence between preferred and experienced patient roles predicts patient satisfaction and low levels of regret. Therefore, prior to assisting patients in adopting a certain role, their individual circumstances should be taken into consideration.
3. It is important that cancer patients perceive the emotional support, care, and appreciation of their personal values from their health providers.

Contemplating the literature, the progress that has been made to study post-treatment patient regret in relation to SDM is undoubtful. Nevertheless, we believe that further research can provide more in-depth, nuanced findings. Deriving from the gaps in the current knowledge, we provide three considerations for future research focusing on SDM and patient regret:

1. Prospective studies on SDM and patient regret may produce more robust findings. Most of the previous research has retrospectively assessed the predicting factors of regret. Assessing these variables in the post-treatment phase may be problematic since in an attempt to rationalize their regret, regretful patients might falsely attribute it to SDM variables. To prevent patient recall bias, we suggest measuring patient-perceived SDM variables prior to the treatment. In Table 1, we propose some measurement tools potentially useful for this goal.
2. Even though SDM in its core definition involves both patients and clinicians, previous literature mostly investigated only patients’ perception of SDM process in relation to regret. Our knowledge of physician-reported variables in this regard is scarce. This is problematic, as the rights and responsibilities of both physicians and patients are inseparable elements of the physician–patient relationship. It may be particularly informative to study clinicians’ attitudes, communication style, and decision-making role preferences to obtain the holistic viewpoint of the dynamics of the patient-physician relationship and its effects on patient regret.
3. There may be aspects of SDM that cannot be grasped by patient or clinician self-reports, nonetheless, implicitly having an effect on patient regret. To address this issue, we suggest applying a more impartial assessment, for instance, analysis of the medical consultation recordings conducted by a third person (researcher), uninvolved in SDM process.

In summary, previous research has focused on the key aspects of SDM that predict regret. In an attempt to foster even more informative, multidimensional, and robust research, we made some methodological suggestions for future studies. Overall, the evidence suggests that adopting an individualized approach and providing patients with both information and emotional support is essential. This empirical knowledge is of high importance as it serves as the guideline for the clinicians, involved in the SDM process.

## Data Availability

Not applicable.

## Abbreviations

DA: Decision aid
SDM: Shared decision-making
